# International guideline development for the determination of death

**DOI:** 10.1007/s00134-014-3242-7

**Published:** 2014-03-25

**Authors:** Sam D. Shemie, Laura Hornby, Andrew Baker, Jeanne Teitelbaum, Sylvia Torrance, Kimberly Young, Alexander M. Capron, James L. Bernat, Luc Noel, and The International Guidelines for Determination of Death phase 1 participants, in collaboration with the World Health Organization

**Affiliations:** Canadian Blood Services and Division of Critical Care, Montreal Children’s Hospital, McGill University Health Centre, 2300 Tupper Street room C-806, Montreal, QC H3H 1P3 Canada

**Keywords:** Death, Brain death, Circulatory death, Cardiac death, Organ donation, Brain injury, Cardiopulmonary resuscitation, Autoresuscitation

## Abstract

**Introduction and Methods:**

This report summarizes the results of the first phase in the development of international guidelines for death determination, focusing on the biology of death and the dying process, developed by an invitational forum of international content experts and representatives of a number of professional societies.

**Results and Conclusions:**

Precise terminology was developed in order to improve clarity in death discussion and debate. Critical events in the physiological sequences leading to cessation of neurological and/or circulatory function were constructed. It was agreed that death determination is primarily clinical and recommendations for preconditions, confounding factors, minimum clinical standards and additional testing were made. A single operational definition of human death was developed: ‘the permanent loss of capacity for consciousness and all brainstem functions, as a consequence of permanent cessation of circulation or catastrophic brain injury’. In order to complete the project, in the next phase, a broader group of international stakeholders will develop clinical practice guidelines, based on comprehensive reviews and grading of the existing evidence.

## Introduction

Philosophical, religious, and cultural differences in the concept of death and ways it is defined make discussions of the subject very complex. Advances in medicine and technology that have made it possible to support, repair, or replace failing organs challenge commonly held notions of life and death. The lack of understanding or even awareness among the public [1] and health professionals [2] and the emotionally charged nature of the subject further complicate discussions. Finally, the determination of death is deeply entwined with organ transplantation. The growing disparity between the demand for and the availability of transplantable organs is a public health concern globally [3]. In comparison to bridge technologies for end-stage organ failure, such as kidney dialysis [4] and ventricular assist devices [5], organ transplantation is life-preserving, life-enhancing, and cost-effective. Yet, when the supply of transplantable organs falls short of demand, unethical and illegal behaviours sometimes occur, including commercial organ trade that victimizes the vulnerable [6], human killing as a source of transplantable organs [7], and violations of the allocation system [8].

While the public supports organ donation after death, its credibility requires clarity in practice and policy. Depending on the country and related professional societies, guidelines may or may not exist for the determination of death by neurological and/or circulatory criteria, and when guidelines are in place, they have not always been implemented in a manner that alleviates concerns about the legitimacy of deceased organ donation. The situation is complicated by variations related to the stage of development of a country’s deceased donation program and more generally by the significant diversity in health services and standards of care worldwide. The World Health Organization (WHO) and The Transplantation Society (TTS) have received requests from a number of countries to address these gaps and to provide guidance to inform clinical practices and health policy for death determination as part of an international effort in support of the Istanbul Declaration [9], the WHO Madrid Resolution [10], and the WHO Guiding Principles on Human Cell, Tissue and Organ Transplantation, approved by the World Health Assembly in 2010 [11]. An international consensus on the determination of death could provide a number of benefits including promoting evidence-based practices, protecting the rights of both patients and health care professionals, improving public and professional confidence in the process of deceased donation, and increasing the number of organs obtained in an ethically legitimate fashion.

## Methods

On 30–31 May 2012, an invitational forum sponsored by Health Canada and Canadian Blood Services in collaboration with the WHO was held in Montreal, Canada, as part of the planning, scoping, and needs assessment phase in the process of guideline development (see “Appendix 1” for forum committees and participants). The 32 invited participants included delegates from a broad range of national and international professional societies involved with death determination in adults and children in acute care settings. Prior to the forum, participants were provided with comprehensive background materials, including a selected bibliography of peer-reviewed articles related to death determination, a literature review on definitions of death, and a draft lexicon of medical terminology relevant to death determination policy and practice. Each section of the meeting began with an expert speaker who provided historical information, reviewed baseline knowledge, and supplied his or her perspective on current issues and controversies. After each presentation, participants asked questions of the expert and discussed the presentation with the entire group. Reference sheets that offered condensed summaries of existing evidence were distributed, and the participants were then divided into groups where they held extensive discussions about a “challenge question” relevant to the topic of the session. When the participants reconvened in the plenary, each group’s results were discussed, and the collective outputs were reworked until consensus was reached. Consensus was defined as achieving substantial agreement, manifested by all participants indicating that they agreed with or could accept a conclusion, which they would support both within and outside the meeting.

For the purposes of this forum, death was fundamentally considered a biological event. While it was respectfully recognized that legal, ethical, cultural, and religious perspectives on death can impact the utility of recommendations in the field, these perspectives were not topics for debate or discussion at this initial meeting. Table 1 highlights the common terminology that was agreed upon to support clarity and precision in the language used throughout this forum and for subsequent phases of work. The planning committee worked with participants to identify critical events that comprise the dying sequence, with the understanding that dying is a process whereby biological/physiological functions cease. Death is an event in that dying process, a point when the person can be determined to have died. The forum identified the tests required for the minimum acceptable clinical standard for determining death in adults and children and recommended additional testing beyond the minimum standard. A consensus on an operational definition of human death was then derived.

**Table 1:** Participants agreed to the following terminology, in order to improve the clarity of discussions and debate, for use during and subsequent to this forum

Term	Definition
Activity	Physiologic properties of cells and groups of cells that can be measured by laboratory means
Asystole—electrical	A condition characterized by the absence of electrical, and hence mechanical, activity of the heart, resulting in the absence of contractions of the myocardium and cardiac output/anterograde blood flow
Asystole—mechanical	The absence of effective contractions of the myocardium and no cardiac output/anterograde blood flow. May occur in the presence of an organized or disorganized electrocardiac rhythm, e.g. pulseless electrical activity
Autoresuscitation	The spontaneous resumption of heart contractions causing anterograde circulation that is not induced by cardiopulmonary resuscitation or other external assistance. Examples of heart function such as effective contractions of the myocardium leading to anterograde flow of blood through the aorta and arterial system should be distinguished from examples of heart activity such as atrial natriuretic hormone release or residual pulseless electrical activity
Brain death	Diagnosis and confirmation of death based on the irreversible cessation of functioning of the entire brain, including the brainstem (this forum supports the movement away from this traditional and imprecise terminology in favour of the cessation of neurological function)
Brainstem death	Diagnosis and confirmation of death based on the irreversible cessation of functioning of the brainstem, predominantly but not exclusively secondary to a supratentorial brain injury (this forum supports the movement away from this traditional and imprecise terminology in favour of the cessation of neurological function)
Cardiac arrest	The abrupt cessation of circulation of the blood due to failure of the heart to contract effectively. Also known as cardiorespiratory arrest, cardiopulmonary arrest, or circulatory arrest
Catastrophic brain injury leading to death	Etiologies of high severity that are common causes of brain death, such as, but not limited to, traumatic brain injury, cerebrovascular accidents, and hypoxic-ischaemic encephalopathy after resuscitated cardiac arrest Other forms of catastrophic brain injury that have any degree of residual clinical brain or brainstem function are not under consideration and may include persistent vegetative states, permanent vegetative states, anencephaly, or those conditions related to the historical concept of higher brain (cortical) death
Cerebral electrical activity	Electrical activity of the brain, as measured using an electroencephalogram (EEG)
Cessation	Stoppage, termination
Circulation	Anterograde flow of blood through the aorta and arterial system
Circulatory death determination	Diagnosis and confirmation of death based on circulatory criteria. Also known as death after cardiac arrest, or death after cardiocirculatory arrest, or death after circulatory-respiratory determination (this forum supports the movement away from this traditional and imprecise terminology in favour of the cessation of circulatory function)
Clinical	Based on direct, measurable observation or examination of the patient
Coma	Prolonged absence of wakefulness, awareness, and the capacity for sensory perception or responsiveness to the external environment
Confounding conditions	Circumstances during which a diagnostic test may become unreliable and require repetition over time or application of an alternative test
Consciousness—loss of capacity for	Lack of current or any future potential for awareness, wakefulness, interaction and capacity for sensory perception of, or responsiveness to the external environment
Criteria—minimum	Refers to the lowest acceptable standard. The standard recommended by this forum sets the minimum clinical criteria
Dead donor rule	A principle governing deceased donation practices stating that vital organs should only be taken from dead patients and, correlatively, living patients must not be killed by organ retrieval. This rule does not apply to, nor preclude, living donation of non-vital organs
Death	The moment in time during the dying process when the individual passes from the state of being alive to that of being dead
Death—concept of	An abstract, unprovable explanation of death, generally based on religious, spiritual, or philosophical beliefs
Death—declaration of	The point in time at which a health professional, having determined that an individual is dead, formally states this finding
Death—operational definition of	Biomedical criteria that describe the state of human death
Death—determination of	Processes and tests required to diagnose death in accordance with established criteria
Disintegration	Loss of intactness, solidness, or cohesion. Such loss can apply to function or to matter (tissues, etc.)
Dying	The process whereby biological/physiological functions cease, thus moving from the state of being alive to that of being dead
ECMO	Extracorporeal membrane oxygenation/extracorporeal oxygenation and circulation of blood deployed for life-threatening lung or heart–lung failure
Electromechanical dissociation	A form of mechanical asystole. A rhythm frequently encountered during cardiac arrest, characterized by organized electrical activity without circulation, traditionally measured by the absence of a palpable pulse or pulsatile arterial blood pressure
Fixed dilated pupils	Pupils in mid-position or greater and unreactive to light
Function	In the context of organs, the primary and fundamental purpose of that organ that can be assessed by observation and examination and is necessary for sustained life. Function should be distinguished from activities, as defined by physiologic properties of cells and groups of cells that can be measured by laboratory means. Examples of brain function such as the capacity for consciousness or ability for unassisted breathing should be distinguished from examples of brain activity such as posterior pituitary antidiuretic hormone release or residual nests of neuronal electrical activity
Integration	Combined or coordinated separate elements that provide a harmonious, interrelated whole; organized or structured so that constituent units function cooperatively
Irreversible	Pertaining to a situation or condition that will not or cannot return or resume. In the context of death determination, there are variable definitions including: 1.Loss of function or a condition that cannot be restored by anyone under any circumstances at a time now or in the future 2.Loss of function or a condition that cannot be restored by those present at the time 3.Loss of function or a condition that will not resume and will not be restored. Also referred to as permanent
Neuroimaging	Diagnostic brain imaging techniques to identify structural brain injury, e.g. CT scan, MRI
Neurological death determination	Diagnosis and confirmation of death based on neurological criteria
No effective intervention	A therapeutic intervention that is not deployed because it is not effective, not medically indicated under those circumstances, not available or accessible
Mechanical ventilation	Assisted ventilation including bag/mask ventilation, non-invasive support e.g. BiPAP (bilevel positive airway pressure), conventional mechanical ventilation via artificial airway
Refractory to treatment	Does not respond to intervention in a clinically meaningful manner
Permanent	Pertaining to a situation or condition that will not return to its previous state. In the context of death determination, refers to loss of function that will not resume spontaneously and will not be restored through intervention
Preconditions	Patient-related prerequisites that should be fulfilled prior to application of diagnostic tests
Respiratory arrest	Cessation of breathing. In the context of death discussions, this may be primary and lead to a subsequent cardiac arrest, or it may be secondary to the loss of brainstem function
Test	A procedure performed in diagnosis or detection
Test—ancillary	A complementary test or an alternative test to one that otherwise, for any reason, cannot be conducted or is unreliable
Test—clinical	A bedside test typically based on physical examination of the patient, but may include the use of a stethoscope and vital signs monitors
Test—confirmatory	A test performed to confirm a previously conducted test
Test—laboratory	A technical test requiring use of elaborate equipment and medical technologies, e.g. blood testing, diagnostic imaging
Test—supplemental	A test performed in addition to an already conducted test
Unity	The combination or arrangement of parts into a whole
Ventricular fibrillation	A condition in which there is uncoordinated contraction of the cardiac muscle of the ventricles that causes the cessation of circulation. Also referred to as V-fib or VF
Vital function	Necessary for sustained life

## Findings

### Neurological sequence in the dying process

**Fig. 1:**
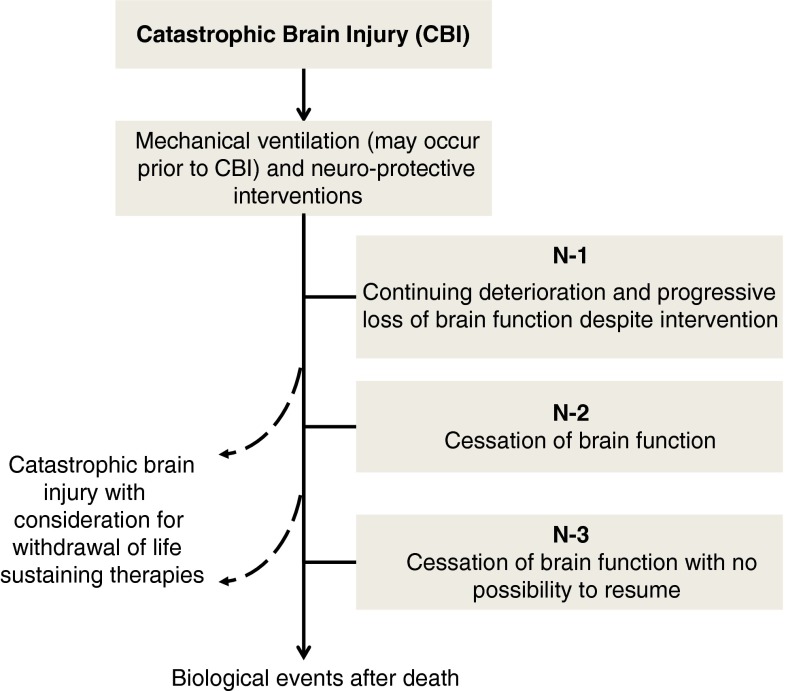
Neurological sequence in the dying process

Forum participants identified several major sequential events in the dying process of patients who have suffered a catastrophic brain injury that will lead to death determined on a neurological basis (such as, but not limited to, traumatic brain injury, cerebrovascular accidents, and hypoxic-ischaemic encephalopathy after resuscitated cardiac arrest) (Fig. 1). This sequence does not apply to patients with anencephaly or with forms of catastrophic brain injury (such as persistent vegetative states) where residual clinical brain or brainstem functions are retained; such patients were not under consideration. Patients entering this sequence are receiving mechanical ventilation; various other neuroprotective interventions (such as hyperosmolar therapy, ventricular drainage, decompressive craniectomy) may have been initiated as well. At N-1, the patient continues to deteriorate in spite of intervention and the treatment team recognizes that the patient may evolve to brain death. At N-2, the deterioration has continued to a point that brain function has ceased. However, at this point it is still possible that brain function could return spontaneously or be restored through intervention. If preconditions are met, confounding factors are absent, and no effective treatment is available or implemented, then by N-3, the brain has ceased functioning and there is no possibility to resume. The patient has died.

Preconditions prior to testing brain function at any point in the sequence include an established etiology; absence of reversible etiologies that would explain the coma; and the absence of hemodynamic shock associated with inadequate oxygenated circulation to the brain [12]. Confounding conditions that may invalidate testing for cessation of brain function include naturally occurring or therapeutic hypothermia; the presence of central nervous system (CNS) depressing drugs that may explain or contribute to coma; high cervical spine injury; acquired (such as severe polyneuropathy) or therapeutic neuromuscular paralysis; locked-in syndromes; and severe acid-base, electrolyte or endocrine abnormalities that may explain or contribute to coma [13]. After agreeing that death is principally established using clinical criteria (defined as diagnostic testing based on direct, measurable observation or examination of the patient; see Table 1), participants came to consensus on the “minimum acceptable clinical standards” to test for the cessation of brain function (Table 2). It was also agreed that the validity of a determination of death depends upon the health care professionals performing the clinical determination and those performing and interpreting ancillary laboratory testing possessing the necessary competencies.

**Table 2:** Minimum acceptable clinical standard and additional tests for death after cessation of brain function in adults and children

	Description	Minimum acceptable clinical standard	Additional testing (beyond the minimum clinical standard)
N-1	Catastrophic brain injury: continuing deterioration and progressive loss of brain function despite intervention	1. Established etiology and/or structural lesion capable of causing death by neurological criteria 2. Reduced consciousness (as measured by GCS 3–5 or FOUR score) 3. Evidence for progressing loss of brainstem function	1. Neuroimaging that explains the severity of brain injury 2. Repetition of clinical exams with trends 3. Demonstration of elevated intracranial pressure (ICP) by monitoring
N-2	Cessation of brain function	1. Coma (excluding spinal cord mediated reflexes) 2. Absence of brainstem reflexes: •Pupils mid-position or greater and absent pupillary light reflex (fixed dilated pupils) •Corneal •Gag/pharyngeal •Cough/tracheal •Vestibulo-ocular (‘cold caloric’) •Loss of central drive to breathe NB: performance of apnoea testing should be reserved as the last test of brainstem function	None: cessation of brain function is a clinical determination
N-3	Cessation of brain function with no possibility to resume	1. Preconditions fulfilled 2. Confounding conditions excluded or addressed 3. Refractory to all applied interventions 4. Intervention not available or indicated	1. Repetition of the minimum clinical standard examination 2. Ancillary laboratory tests e.g. •Demonstration of brain blood flow or perfusion to be absent •Refractory intracranial hypertension as measured by ICP monitoring •Transcranial Doppler consistent with absent net flow velocity •Electrodiagnostic testing (e.g. EEG, absent evoked potentials)

### Circulatory sequence in the dying process

**Fig. 2:**
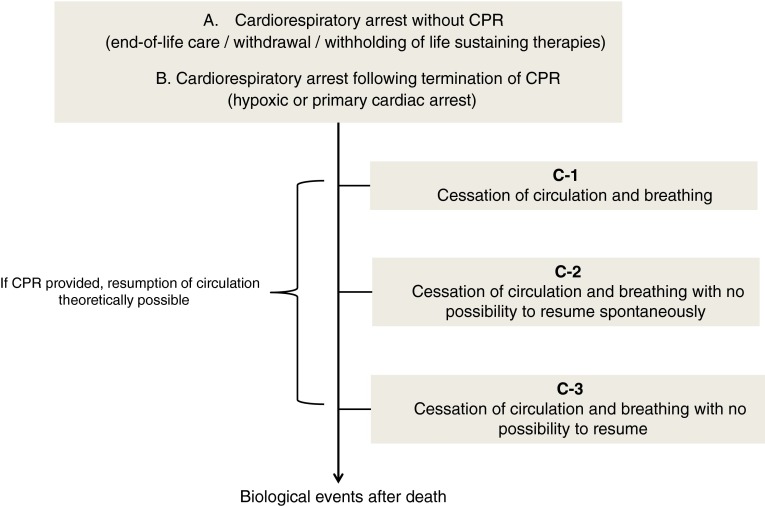
Circulatory sequence in the dying process

Forum participants identified several major sequential events in the dying process of patients who suffer a circulatory arrest (Fig. 2). In situation A, the patient has had a cardiac arrest but no CPR intervention, either because CPR was not medically indicated or the patient (or surrogate decision maker) declined it; this would include terminally ill patients whose end-of-life care involves limiting or withdrawing life-sustaining therapies. At C-1, the patient’s circulation and breathing stop. After a certain time period (between 2 and 5 min, based on expert consensus) [14], autoresuscitation-the spontaneous, unassisted resumption of circulation-is no longer a possibility under these conditions (C-2). There is currently no published evidence demonstrating autoresuscitation under these conditions [15–17]. Since no interventions will be made to attempt to restore circulation, cessation of breathing and circulation is permanent and the patient may be determined to be dead (C-2 and C-3 occur at the same time). In situation B, CPR has been used in an attempt to restore circulation and respiration but has been terminated because the patient cannot be revived. Once the time interval when autoresuscitation is possible has passed, cessation of breathing and circulation is permanent and the patient may be determined to be dead. International practice varies with regard to this time interval between C-1 and C-2. The most common waiting period is 5 min with a range from 2 to 10 min [18]. The participants came to consensus on the ‘minimum acceptable clinical standards’ to test for the cessation of circulatory function (Table 3).

**Table 3:** Minimum acceptable clinical standard and additional tests for death after cessation of circulatory function in adults and children

	Description	Minimum acceptable clinical standard	Additional testing (beyond the minimum clinical standard)
C-1	Cessation of circulation and breathing	1. Absent palpable pulse 2. Absent breath sounds 3. Absent heart sounds 4. Absent respiratory effort or chest wall motion 5. Loss of pulsatile arterial blood pressure by non-invasive measurement 6. Coma and fixed dilated pupils 7. Electrical asystole is not required (pulseless electrical activity is acceptable)	1. Loss of pulsatile arterial blood pressure by arterial line monitoring 2. Absence of anterograde blood flow through the aortic valve on echocardiography 3. Isoelectric ECG 4. Absence of pulse by Doppler NB: oxygen saturation pulse oximetry is an unreliable indicator of absence of pulsatile circulation
C-2	Cessation of circulation and breathing with no possibility to resume spontaneously	1. The persistence of C-1 criteria over a period of time as confirmed by continuous observation and intermittent confirmation including repetition of this evaluation at the end of the period. The time period required is 2–5 min 2. When breathing and circulation cease following terminated CPR, the time period to reach the point of “no possibility to resume spontaneously” is 2–10 min	1. Use of the same tests for a higher clinical/laboratory standard for C-1 applied after the time interval required to progress from C-1 to C-2 (2–10 min following termination of CPR)
C-3	Cessation of circulation and breathing with no possibility to resume	1. When CPR will not be provided (patient fulfils criteria for not providing CPR) C-3 occurs at the moment of C-2 2. Following termination of CPR, including a decision not to reinstitute CPR, C-3 and C-2 occur at the same time	1. Nothing in addition to those tests required for C-2

### Integrated neurological and circulatory sequence in the dying process

**Fig. 3:**
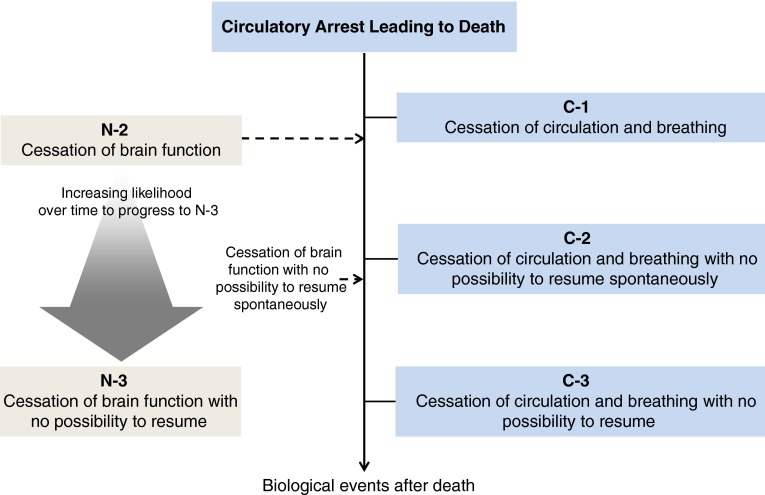
Physiological sequences in the dying process: integrated neurological and circulatory sequence (applies to patients suffering a circulatory arrest)

The inextricable link between circulation and brain function means that the neurological and circulatory sequences integrate at several points in the dying process of patients who have suffered a circulatory arrest (Fig. 3). Once circulation and breathing cease (C-1), there is a short time period between C1 and N2 (<20 s) during which brain function ceases (N-2) as evidenced by isoelectric EEG [19–21]. The longer the time period without oxygenated circulation to the brain (N-2 to N-3) the progressively higher likelihood that the cessation of brain function is irreversible, even if oxygenated circulation can be re-established (either spontaneously or through intervention). The precise time period for the complete cessation of brain function to be non-resuscitable (through intervention) is unresolved.

### Operational definition of human death

After reviewing the historical taxonomy and definitions of death, and the neurologic and circulatory sequences in the dying process that had been previously discussed and refined, participants came to consensus on an operational definition of human death, that is, a practical and quantifiable description of the state of human death based on measurable and observable biomedical standards. Forum participants agreed on the following operational definition of death:

*Death is the permanent loss of capacity for consciousness and all brainstem functions. This may result from permanent cessation of circulation or catastrophic brain injury. In the context of death determination, ‘permanent’ refers to loss of function that cannot resume spontaneously and will not be restored through intervention* [22].

Participants supported avoiding use of anatomically based terms such as “brain death” or “cardiac death” that erroneously imply the death of that organ and confuse the general public, health professionals, and policymakers (organisms die, while organs cease functioning). Our operational definition is based on the cessation of function (the primary and fundamental purpose of an organ that can be assessed by observation and examination and is necessary for sustained life) rather than activities (physiologic properties of cells and groups of cells that can be measured by laboratory means). While the forum participants understood that the overwhelming majority of deaths in the world occur after circulation has ceased, and many occur outside health care settings, death determination must focus on the centrality of brain function. Death is a single phenomenon based on permanent cessation of brain function (loss of capacity for consciousness and brainstem reflexes), which occurs along two pathways: (1) permanent absence of circulation or (2) subsequent to a catastrophic brain injury, each discerned through a specific set of medical criteria and clinical and laboratory tests—two entrances, one end point.

## Conclusions

This report describes the initial phase in an international process to develop guidelines and agree upon an operational definition for determining death, based on the cessation of neurological and circulatory functions. Plenary discussions resulted in consensus on seven key areas:

1. Death is determined primarily using clinical criteria based on direct observation or examination of the patient, once preconditions have been fulfilled and confounding conditions excluded.
2. The physiological sequences by which circulatory and neurological functions cease, leading up to the moment of death, were set forth to clarify the critical events that occur following a catastrophic injury or illness.
3. Clinical tests that constitute the minimum clinical standard for the determination of death were defined for both the neurological and the circulatory sequences. Preconditions and confounding conditions that may impede or invalidate death diagnosis were also specified.
4. Certain ancillary and/or complementary laboratory tests may be useful in situations in which clinical testing cannot be performed or when confounding or special conditions are present; limitations to using certain of these tests in death determination mean that further research is required to ensure their reliability.
5. A set of precise terminology, which aims to improve clarity in death determination discussions and debate, was agreed upon.
6. An operational definition of human death, based on measurable biomedical standards, was proposed. Participants supported avoiding use of anatomically based terms such as “brain death” or “cardiac death” that erroneously imply the death of an organ. Emphasis was placed on the cessation of neurological or circulatory function, and the centrality of brain function for determination of death. “Death occurs when there is permanent loss of capacity for consciousness and loss of all brainstem functions. This may result from permanent cessation of circulation or catastrophic brain injury. In the context of death determination, ‘permanent’ refers to loss of function that cannot resume spontaneously and will not be restored through intervention.” This definition is based on the cessation of function (the primary and fundamental purpose of an organ that can be assessed by observation and examination and is necessary for sustained life) rather than activities (physiologic properties of cells and/or groups of cells that can be measured by laboratory means).
7. In order to complete the elaboration of an operational definition of human death that could be used in countries around the world, a broader group of international stakeholders will be needed to develop clinical practice guidelines, based on comprehensive, systematic reviews and grading of existing evidence.

## Appendix 1: Forum committees, speakers, and organizations

Forum planning committee: Dr. Sam Shemie (forum chair), Dr. Andrew Baker, Ms. Laura Hornby, Ms. Dorothy Strachan, Dr. Jeanne Teitelbaum, Ms. Sylvia Torrance, Ms. Kimberly Young.

Forum international advisory committee: Dr. Sam Shemie (forum chair), Dr. James Bernat, Professor Alexander Capron, Dr. Frank Delmonico, Dr. Luc Noel.

Forum research group: Dr. Andrew Baker, Ms. Laura Hornby, Ms. Dorothy Strachan, Dr. Jeanne Teitelbaum, Dr. Tong Kiat Kwek, Dr. Alexander Manara.

Expert speakers: Dr. Sam Shemie—Challenge address; Dr. Luc Noel—International perspectives; Dr. Eelco Wijdicks—Death and the brain; Dr. James Bernat—Death and the circulation; Prof. Alexander M. Capron—Historical review of the definition of death; Dr. Charles Sprung—Building global agreement around complex practices; Dr. Margaret Harris—WHO guidelines development process.

Participating organizations: Canadian Blood Services; Australia and New Zealand Intensive Care Society; Canadian Critical Care Society; European Society of Intensive Care Medicine; International Federation of Emergency Medicine; International Pan Arab Critical Care Medicine Society; International Society for Organ Donation and Procurement; Neurocritical Care Society; Society of Critical Care Medicine; United Kingdom Faculty of Intensive Care Medicine; University of Ottawa Loeb Chair and Research Consortium in Organ and Tissue Donation; World Federation of Neurology; World Federation of Neurosurgical Societies; World Federation of Pediatric Intensive and Critical Care Societies; World Federation of Societies of Intensive and Critical Care Medicine; World Health Organization.

## Appendix 2: International Guidelines for Determination of Death phase 1 participants

Dr. Tamer Abdelhak, International Pan Arab Critical Care Medicine Society; Dr. Sadek Beloucif, France; Dr. Mohammed Salah Ben Ammar, Tunisia; Dr. Andrew Baker, Canadian Critical Care Society; Dr. James L. Bernat, Department of Neurology, Geisel School of Medicine at Dartmouth; Dr. Peter Black, World Federation of Neurosurgical Societies; Dr. Thomas Bleck, Society of Critical Care Medicine; Dr. Romuald Bohatyrewicz, Poland; Prof. Alexander M. Capron, Professor of Law and Medicine, University of Southern California; Dr. Giuseppe Citerio, European Society of Intensive Care Medicine; Prof. Geoff Dobb, Australia and New Zealand Intensive Care Society; Laura Hornby, Loeb Chair and Research Consortium in Organ and Tissue Donation; Dr. Waleed Jasim, International Pan Arab Critical Care Medicine Society; Dr. Edgar Jimenez, World Federation of Societies of Intensive and Critical Care Medicine; Dr. Günter Kirste, International Society for Organ Donation and Procurement; Dr. Niranjan Kissoon, World Federation of Pediatric Intensive and Critical Care Societies; Dr. Tong Kiat Kwek, Singapore; Dr. Alexander Manara, UK Faculty of Intensive Care Medicine; Dr. Luck Noel, World Health Orgnization; Dr. Sam D. Shemie; Dr. Mikhail Sinkin, Moscow Mobile Neurodiagnostic Group, Russia; Prof. Charles L. Sprung, Israel; Dr. Gene Sung, Neurocritical Care Society; Dr. John Tallon, International Federation of Emergency Medicine; Dr. Jeanne Teitielbaum, Canada; Ms Sylvia Torrance, Canadian Blood Services; Dr. Eelco Wijdicks, USA; Dr. G. Bryan Young, World Federation of Neurology.
